# A Mobile App to Increase Fruit and Vegetable Acceptance Among Finnish and Polish Preschoolers: Randomized Trial

**DOI:** 10.2196/30352

**Published:** 2022-01-04

**Authors:** Henna Vepsäläinen, Essi Skaffari, Katarzyna Wojtkowska, Julia Barlińska, Satu Kinnunen, Riikka Makkonen, Maria Heikkilä, Mikko Lehtovirta, Carola Ray, Eira Suhonen, Jaakko Nevalainen, Nina Sajaniemi, Maijaliisa Erkkola

**Affiliations:** 1 Department of Food and Nutrition University of Helsinki Helsinki Finland; 2 Faculty of Psychology University of Warsaw Warsaw Poland; 3 School of Applied Educational Science and Teacher Education University of Eastern Finland Joensuu Finland; 4 Institute for Molecular Medicine Finland University of Helsinki Helsinki Finland; 5 Folkhälsan Research Center Helsinki Finland; 6 Department of Education University of Helsinki Helsinki Finland; 7 Faculty of Social Sciences Tampere University Tampere Finland

**Keywords:** gamification, intervention, behavior change, health game, games for health, smartphone app, mobile phone

## Abstract

**Background:**

Early childhood education and care (ECEC) centers are ideal venues for food education. As smartphones and tablets are becoming increasingly popular in ECEC centers, technology can be used to deliver such pedagogical content. Evidence suggests that video games can affect fruit and vegetable (FV) consumption among 9- to 12-year-old children, but studies among preschoolers are scarce.

**Objective:**

This paper describes the development of the Mole’s Veggie Adventures app and its effectiveness in increasing FV acceptance among Finnish and Polish preschoolers aged 3 to 6 years.

**Methods:**

A multiprofessional team created an app to be used in ECEC centers in groups of 3 to 10 children. The app aimed to increase vegetable acceptance, and it was built using elements that support the development of self-regulation and social skills. Altogether, 7 Finnish and 4 Polish ECEC centers participated in the study. Before randomization, parents reported background factors and their children’s willingness to taste different FVs. The ECEC professionals in the intervention arm were instructed to use the app at least once a week during the 3- to 4-week intervention period. The main outcomes in this unblinded, cluster-randomized study were FV acceptance and relative FV acceptance. The first was calculated as a sum variable describing the children’s willingness to taste 25 different FVs, the second as FV acceptance divided by the number of FVs served. We used analysis of covariance to compare the FV acceptance and relative FV acceptance scores between the intervention and control groups at follow-up.

**Results:**

A total of 221 children were included in the analysis. At follow-up, the intervention group (115/221, 52%) had higher FV acceptance scores (baseline adjusted difference of mean 7.22; 95% CI 1.41-13.03) than the control group (106/221, 48%). The intervention effect was parallel for relative FV acceptance scores (baseline adjusted difference of mean 0.28; 95% CI 0.05-0.52).

**Conclusions:**

The Mole’s Veggie Adventures app has the potential to increase FV acceptance among preschoolers and can be a valuable tool in supporting food education in ECEC centers. Furthermore, the app can be feasibly incorporated into preschool routines in countries with different educational environments.

**Trial Registration:**

ClinicalTrials.gov NCT05173311; https://tinyurl.com/4vfbh283

## Introduction

### Background

Most European children do not consume the recommended amount of fruit and vegetables (FVs) [1,2]. Among European countries, Poland and Finland face the same challenges. For instance, there seems to be a large proportion of both Polish and Finnish preadolescents who do not eat FVs daily [3]. Studies among Polish preschoolers are scarce, but in a 2011 report, the proportion of Finnish 6- to 8-year-olds consuming the recommended amount of FVs was less than 5% [4]. More recent studies have observed average consumption to be closer to recommendations, but the consumption of vegetables seems to be lower than that of fruit [5,6]. Indeed, children tend to prefer sweet tastes, as observed in fruits, compared with bitter-tasting foods, such as vegetables [7], and need more taste exposures to accept new vegetables [8]. However, as reassuring evidence suggests that repeated taste exposure and even exposure to picture books can help children to learn to enjoy vegetables [7-9], early childhood is a significant phase to support the formation of healthy eating habits among children.

In both Poland and Finland, most 3- to 6-year-olds attend early childhood education and care centers (ECECs) [10,11]. The general aim of the Finnish curriculum for ECEC is to strengthen skills related to children’s well-being [12], such as self-regulation skills, which refer to the ability to monitor and manage emotions and behaviors. Reinforcing self-regulation skills in childhood is important because they are associated with health outcomes later in life [13-15]. Moreover, self-regulation skills are also linked to health behaviors because, for instance, eating is regulated according to internal cues of hunger and fullness [16]. Both the Polish and Finnish recommendations for ECEC encourage ECEC professionals to support the development of self-regulation in eating and to promote a positive attitude toward food and eating [17,18]. Hence, food education is part of the pedagogically guided activities and holistic learning about well-being. However, ECEC professionals lack concrete, age-appropriate, effective, yet appealing tools for food education.

### Objective

Mobile devices, such as smartphones and tablets, are ubiquitous and increasingly used at ECEC centers [19]; thus, food education can be delivered using technology. As ECEC centers should provide children with equal possibilities to familiarize themselves with technology and to practice responsible use of digital devices [12], the use of technology per se is beneficial. Furthermore, video games can trigger feelings of joy, intense participation, social interaction, and pleasure [20,21], and their educational use is considered promising [22]. Vast numbers of educational games and apps are already available through the digital distribution platforms Google Play and the App Store, and some of these even focus on food education. Studies reporting on the use of digital games and food-related outcomes are scarce and mostly concentrate on negative outcomes, such as an increase in fast food consumption due to advergaming exposure [23,24]. However, evidence from the United States suggests that video games can positively affect FV intake among 9- to 12-year-olds [25-27]. Moreover, a mobile app including vegetable-based activities has been shown to increase liking and consumption of vegetables among 3- to 6-year-old children in the United Kingdom [28]; however, educational games targeting ECEC environments are lacking. To fill this gap in knowledge, this paper describes the development of the Mole’s Veggie Adventures app and its effectiveness in increasing FV acceptance among Finnish and Polish preschoolers.

## Methods

### App Development

As part of the European Institution of Innovation & Technology (EIT) Food School Network project and together with a software development company specialized in designing, developing, and implementing serious games and gamified solutions (NordicEdu Oy), we designed and pilot-tested the Mole’s Veggie Adventures mobile app to increase vegetable acceptance among preschoolers. The University of Helsinki team was in charge of the app development, and the educational content of the app was designed by experts in nutrition science, food education, and ECEC. The content was first created in Finnish and later adapted and translated into English and Polish. The design of the app is described in detail in Multimedia Appendix 1. Briefly, the app was designed to be used in ECEC centers in groups of 3-10 children, but the ECEC professionals were encouraged to adjust the contents to fit the current situation in their group. The primary purpose of the app is to familiarize children with FV and increase FV acceptance. Unlike traditional mobile apps, Mole’s Veggie Adventures was built using elements that support the development of self-regulation and social skills. The app consists of 4 seasons, each of which includes 6 FVs. At the time of the intervention, the app listed 6 tasks for each of the vegetables and fruits: (1) Learn, (2) Color, (3) Shape, (4) Taste, (5) Pretend, and (6) Play, and the current version was numbered 0.4.5.0 (7b57516). An updated version of the Mole’s Veggie Adventures app is free of charge and available for download in the App Store and Google Play, and Multimedia Appendix 2 provides an overview of the most important sections of the app. All changes were made after the intervention. Table 1 summarizes the main characteristics of the app.

**Table 1:** Short description (descriptive table modified from the original form [29]) of the Mole’s Veggie Adventures app.

Characteristics	Description
Health topics covered	Food behavior (especially acceptability and consumption of FVs^a^); food education
Targeted age group and environment	3- to 6-year-olds; preschool groups
Short description of the game idea	The main character in the game is the Mole, who moves around in a vegetable patch. The game is divided into seasons, each of which contains 6 FVs. The children can familiarize themselves with the FVs by completing different tasks in a group. For each FV, there are adult-led tasks to be completed in groups. In addition, the game includes a Taste Bank, which can be used to record the number of FVs tasted by the group, and Mini-Games, which can be played individually or in pairs. Multimedia Appendices 1 and 2 describe the contents of the game in more detail
Target players	Individual Dyad Small group
Guiding knowledge or behavior change theories, models, or conceptual frameworks	Interactive tailoring (the ECEC^b^ professionals can adjust the tasks to be suitable for their group); role-playing (the players can learn from each other and from the ECEC professionals); goal setting and social cognitive theory (the group can decide to taste new vegetables together); learning through play (social interaction, motor skills, self-regulation etc.)
Intended health behavior change	Increase in FV acceptance
Clinical or parental support needed?	ECEC professionals or parents needed in the adult-led sections; mini-games can be played without adults
Data shared with parent or clinician?	No
Type of game	Active Role-playing Educational
Game platforms needed to play the game	Smartphone Tablet
Recommended play time	30 min at a time; minimum of 1-2 times a week

^a^FV: fruit and vegetable.

^b^ECEC: early childhood education and care.

### Recruitment

To test the effectiveness of the app, we conducted a feasibility study in 2 countries, Finland and Poland, between September and November 2019. On the basis of the literature regarding pilot trial sample size estimation [30], we aimed to recruit 100 children from both countries. Owing to differences in the ECEC systems and cultural environment, we describe the recruitment separately for the 2 countries. In Helsinki, Finland, 12 ECEC center directors were contacted and asked to participate in the study. Of these, 33% (4/12) declined (2 ECEC centers had a busy schedule, 1 did not have enough resources to participate, and in 1 ECEC center, the ECEC professionals were not enthusiastic about the study). In addition, one director could not be reached by email or phone. Thus, 14 groups from 7 public ECEC centers (58% of those invited) agreed to participate in the study. From the consenting groups, we invited all children to participate in the study. Informed consent was requested from legal guardians (later referred to as parents) via the ECEC groups, and the parents of 56% (130/232) of children invited provided their consent to participate in the study. The study was approved by the Education Division of the City of Helsinki, and the University of Helsinki Ethical Review Board in Humanities and Social and Behavioral Sciences deemed the study to be ethically acceptable (Statement 35/2019).

In Poland, the heads of 4 ECEC centers agreed to participate in the study. The study was carried out in 1 public ECEC center in the countryside (Wilczyn) and 2 public (Międzylesie-Warsaw, Piaseczno-Warsaw) and 1 private (Kobyłka-Warsaw) ECEC center in large urban agglomerations. The University of Warsaw’s research team organized meetings for parents in each ECEC center. The aims of the meetings were to introduce the goals of the study and to present the educational content of the intervention to the ECEC professionals and parents of the participating children. The parents received informed consent forms in the meetings, and of 213 who were invited, the parents of 196 (92%) children provided their consent to participate in the study. The study procedure was evaluated and approved by the Ethics Committee of the Faculty of Psychology at the University of Warsaw.

### Background Characteristics

At baseline, the parents of the participating children completed questionnaires regarding background factors and their children’s FV acceptance. They reported the child’s gender and birthdate as well as the number of children living in the same household. The number of children living in the same household was categorized into 3 groups: 1 child, 2 children, and 3 or more children. In addition, the parents indicated whether the child had any vegetable- or fruit-related food allergies. The parents reported their highest educational level using 6 predefined response options (comprehensive school, upper secondary school, vocational school, bachelor’s degree, master’s degree, and licentiate or doctoral degree), which were categorized into 3 groups: low (comprehensive, upper secondary, or vocational school), middle (bachelor’s degree), and high (master’s degree or higher).

### Outcomes

The parents filled in a questionnaire listing 25 vegetables and fruits and inquiring whether these had been offered to the child during the past 4 weeks and how the child reacted to those that had been served. All the listed vegetables and fruits were introduced in the app. The answer options were 0=*was not offered during the past four weeks*, 1=*refused to touch food*, 2=*touched food but did not put in/near mouth*, 3=*put food to lips but not in mouth*, 4=*put food in mouth but spat out/did not eat*, and 5=*ate food*. A similar questionnaire was used earlier in a UK study examining toddlers’ willingness to taste different foods [8]. For each participant, we calculated an FV acceptance score by summing the answers to each of the 25 vegetable and fruit items, with higher scores indicating a higher FV acceptance (theoretical range 0-125). We also calculated the number of FVs served during the past 4 weeks (range 0-25) and used this information to create a relative FV acceptance score (range 0-5) by dividing the FV acceptance score by the number of FVs served.

### Intervention and Control Arms

After the parents of the participating children had filled in the baseline questionnaires, the participating ECEC centers (in Finland) or groups within the ECEC centers (in Poland) were randomly allocated into intervention and control arms. In Finland, 4 ECEC centers with 7 groups were randomized into the intervention arm, whereas 7 groups from 3 ECEC centers were in the control arm. In Poland, groups within each ECEC center were evenly randomized into the intervention (5 groups from 4 ECEC centers) and control arms (5 groups from 4 ECEC centers). The study was not blinded. Researchers visited the intervention arm groups and introduced the app to the ECEC professionals. The ECEC professionals received a printed guide, which contained instructions and information about the app, and a PDF version of the guide was also available through the app. The ECEC professionals were instructed to use the app with a tablet computer at least one to two times a week during the intervention period (3-4 weeks) and to record the number of tasks completed by their group in a logbook. In addition, we recommended that each group focus on at least six vegetables or fruits during the intervention period. The ECEC professionals also provided quantitative and qualitative feedback using a feedback form. Written feedback was used to update the app after the study period. The control arm groups were instructed to continue their normal routines during the intervention period. They were instructed to refrain from introducing any novel food education methods during the intervention period. After the intervention period, follow-up questionnaires inquiring about the children’s FV acceptance were distributed to the parents of the participating children. To treat the intervention and control groups democratically, the app was introduced to the control ECECs after the study period. The trial was not registered because the study did not assess health outcomes and was thus not a clinical trial (World Health Organization defines a clinical trial as “any research study that prospectively assigns human participants or groups of humans to one or more health-related interventions to evaluate the effects on health outcomes” [31]).

### Statistical Analysis

We used an analysis of covariance-type linear model to compare the FV acceptance and relative FV acceptance scores at follow-up. These models were adjusted with baseline FV acceptance score categories (*missing*, *lower than median*, and *median or higher*). To investigate the sensitivity of the results to the choice of the number of baseline categories, we also used baseline FV acceptance scores in tenths (unadjusted FV acceptance score) and sevenths (relative FV acceptance score) in the models.

## Results

Altogether, 67.8% (221/326) of children had data on FV acceptance and relative FV acceptance scores at follow-up and were included in the analyses. The participating children were on average aged 5.0 years (SD 1.2 years). About half of the participants were girls, and slightly more children participated from the Polish than from the Finnish ECEC centers (Table 2). In 55.7% (123/221) of the participating families, at least one of the parents had a master’s degree or higher education, and most of the respondents (the person who filled in the questionnaires on behalf of the child) were mothers.

Table 3 shows the FV acceptance and relative FV acceptance scores at baseline and at follow-up. At follow-up, the FV acceptance score was 78.5 in the intervention group and 72.4 in the control group, whereas the values for relative FV acceptance scores were 3.97 and 3.75, respectively (Table 3). A score of approximately 3 means that on average, the children put the FVs on the lips but not in the mouth, whereas a score of approximately 4 implies that, on average, the children put the FVs in the mouth but did not eat them. When adjusted for baseline FV acceptance score category, participants in the intervention arm scored higher than control participants (Δ estimate 7.22; 95% CI 1.41-13.03). This corresponds to approximately a 10% improvement because of the intervention compared with a control group participant with the same baseline score. Similarly, relative FV acceptance scores at follow-up were, on average, 0.28 higher (+7%) in the intervention arm group than in the control arm group (95% CI 0.05-0.52). Regarding FV acceptance scores, the sensitivity analyses (data not shown) showed a similar and consistently significant intervention effect (Δ estimate 6.38; 95% CI 0.69-12.07), whereas a smaller and borderline significant intervention effect (Δ estimate 0.19; 95% CI −0.03-0.41) was detected for the relative FV acceptance score.

On average, the intervention arm groups used the app 1.9 times/week during the intervention period. The frequency of app use was missing from one group, but the number of completed tasks was as recommended, suggesting sufficient compliance. Furthermore, 17% (2/12) of groups did not use the app as instructed, that is, they used the app less than once a week and did not complete tasks related to at least six FVs. Both groups were from Finnish ECEC centers. The app was typically used in a group of 2-10 children in the Finnish ECEC centers, whereas the usual group size in the Polish ECEC centers was 24-25 children. The most popular FVs chosen by the intervention arm groups were blueberries (11/12, 92% of the groups completed related tasks); lettuce (10/12, 83%); mushrooms (9/12, 75%); kidney, brown, and black beans (9/12, 75%); beetroot (8/12, 67%); and squash (8/12, 67%).

**Table 2:** Description of the study population (N=221).

Characteristics		Total (N=221), n (%)	Intervention (n=115), n (%)	Control (n=106), n (%)
**Gender**				
	Girls	120 (54.3)	67 (58.3)	53 (50.0)
	Boys	100 (45.2)	47 (40.9)	53 (50.0)
	Missing	1 (0.5)	1 (0.9)	0 (0.0)
**Country**				
	Finland	95 (43.0)	50 (43.5)	45 (42.5)
	Poland	126 (57.0)	65 (56.5)	61 (57.5)
**Vegetable or fruit allergy**				
	No	208 (94.1)	108 (93.9)	100 (94.3)
	Yes	12 (5.4)	7 (6.1)	5 (4.7)
	Missing	1 (0.5)	0 (0.0)	1 (0.9)
**Number of children living in the same household**				
	One	56 (25.3)	27 (23.5)	29 (27.4)
	Two	124 (56.1)	62 (53.9)	62 (58.5)
	Three or more	36 (16.3)	22 (19.1)	14 (13.2)
	Missing	5 (2.3)	4 (3.5)	1 (0.9)
**Respondent**				
	Father	27 (12.2)	15 (13.0)	12 (11.3)
	Mother	193 (87.3)	100 (87.0)	93 (87.7)
	Missing	1 (0.5)	0 (0.0)	1 (0.9)
**Parental educational level**				
	Upper secondary school or lower	55 (24.9)	32 (27.8)	23 (21.7)
	Bachelor’s degree or equivalent	39 (17.6)	21 (18.3)	18 (17.0)
	Master’s degree or higher	123 (55.7)	60 (52.2)	63 (59.4)
	Missing	4 (1.8)	2 (1.7)	2 (1.9)

**Table 3:** FV^a^ acceptance and relative FV acceptance scores in the intervention (n=82-115) and control (n=79-106) groups at baseline and at follow-up.

Characteristics			Baseline, mean (SD)		Follow-up, mean (SD)
**FV acceptance score^b^**					
	Intervention group	70.6 (25.5)		78.5 (30.6)	
	Control group	70.2 (25.0)		72.4 (26.2)	
**Relative FV acceptance score^c^**					
	Intervention group	3.84 (1.06)		3.97 (1.03)	
	Control group	3.77 (1.10)		3.75 (1.01)	

^a^FV: fruit and vegetable.

^b^FV acceptance score: sum variable describing willingness to taste the 25 FVs listed; higher score indicates higher FV acceptance (theoretical range 0-125).

^c^FV acceptance score: FV acceptance score divided by the number of FVs served (range 0-5).

## Discussion

### Principal Findings

This paper describes the design and pilot-testing of the Mole’s Veggie Adventures app, which aimed to increase FV acceptance among 3- to 6-year-old preschoolers in Finland and Poland. Our pilot study showed a favorable and meaningful intervention effect; compared with the control arm participants, the participants in the intervention arm had higher FV acceptance scores at follow-up 3-4 weeks after baseline. Thus, the app can be considered an effective food education tool in an early education environment. Earlier studies have shown that video games are effective in increasing FV consumption among 9- to 12-year-old children [25-27], and promising results among preschool aged participants have also been obtained [28]. To the best of our knowledge, the current food education tool is the first to be used routinely in a preschool environment. As healthy food behaviors, such as frequent and diverse FV consumption, are typically adopted in childhood and may track into adulthood [32-34], early childhood is a crucial time to intervene.

### Comparison With Earlier Work

Some serious games emphasize increased knowledge [35], whereas others aim to incorporate multiple theory-driven behavior change techniques, such as tailoring, goal setting, problem solving, and feedback, into a fun and attractive game [36]. In Mole’s Veggie Adventures, no specific behavior change technique was selected, but several of them were included in the game mechanics. For instance, the ECEC professionals were instructed to select those FVs for discussion that they deemed most important for their group. Moreover, the game includes Mini-Games with educational and knowledge-enhancing content. In addition, advergame researchers have described multiple methods that can potentially influence player behavior [37]. Advertising in games can appear at different levels of the game and in many forms, for example, as product placement, background presentation, and engagement via interactivity. In the case of the Mole’s Veggie Adventures app, all the aforementioned expositions of FVs are present, as the product, FVs, appears both in the background and is subject to manipulation itself, potentially enhancing FV acceptance among children. In addition, because of the various types of tasks and activities in the game, the children do not familiarize themselves with FVs only virtually but also in reality. The diverse stimuli with FVs as the main characters may encourage children to become familiar with them. However, the extent to which such an intervention could realistically affect behavior warrants further research.

The Mole’s Veggie Adventures app includes a strong social aspect. It has been shown that ECEC professionals’ opinions may contribute to children’s food consumption [38]. In addition, peers may also act as role models for preschoolers [39]. As our game was used in the ECEC centers in a group of preschoolers, it may have offered opportunities to model—for better or worse—the early educators as well as other children and motivated children to try new FVs. Social interaction provides opportunities for problem solving and peer engagement, which in turn, can cultivate useful skills such as negotiation and cooperation [40]. In addition, approval from the early educator, supporting comments from the group, and the opportunity to boast and present the results of tasks in the group may have been rewarding. Previous serious game research has also suggested that engaging parents—gatekeepers of the home environment [41]—may be critical in changing child behavior [26,27]. Bearing this in mind, we updated the Mole’s Veggie Adventures app after the intervention to better fit both the preschool and home environments (Multimedia Appendices 1 and 2). We also encourage future game designers to consider including a parental component to ensure adult support in all environments relevant to the child.

Fun is an essential part of playing games and can produce intrinsic motivation in players [42]. However, it remains unknown how certain target groups (eg, preschoolers) comprehend and experience fun or how to use fun to design games to bring about larger or more consistent changes in health behaviors [43]. Baranowski et al [42] contemplated the building blocks of fun and suggested that fun in games is probably a combination of interaction, overcoming challenges, making choices, and detecting their consequences without risking oneself, receiving feedback, increasing difficulty through levels of game play, and using personally relevant stories and characteristics in meaningful situations. To ensure that the Mole’s Veggie Adventures app would be perceived as fun, preschoolers participated in the development process (see Multimedia Appendix 1 for details). In addition, the game incorporated elements, such as physical play, invented stories, and adult-led activities, which have been identified as occasions for fun and shared humor among preschoolers [44]. A growing consensus describes play as an intrinsically motivated activity that results in joyful discovery [40]; thus, it is presumable that the app, by covering various forms of play (ie, active physical and pretend play), was indeed perceived as fun by the preschoolers.

Although games can deliver food education in an enticing way, not all behaviors encouraged by games are beneficial. Excessive gaming can evoke negative psychosocial effects [20] and even cause addictive behaviors [45]. Possible adverse effects include increased impulsivity [46], and as impulse control is an element in self-regulation, games can impair the development of self-regulation skills. Poor self-regulation skills in childhood have been linked to diminished social and cognitive outcomes later in life [47] and may also be associated with adverse health outcomes such as overweight and obesity or increased screen time [48-52]. To avoid these pitfalls, the Mole’s Veggie Adventures app was designed to support the development of self-regulation skills. For instance, the app includes elements that require peaceful action and waiting. Moreover, the app is mostly intended to be used with an ECEC professional, whose role as a coregulator is significant in strengthening self-regulation skills [53]. Subdued, mild colors and delicate music allow the child to focus on the educational content and could potentially prevent impulsivity.

### Study Strengths and Limitations

The strengths of the study include testing in 2 countries, Finland and Poland. The 2-country setting allowed us to recruit a larger sample, which in turn, enabled the detection of the intervention effect. Another strength is the random allocation of ECEC centers into the intervention and control arms. Therefore, it is unlikely that the outcome was confounded by uncontrolled variables. Moreover, we examined one specific outcome (FV acceptance) instead of testing for multiple outcomes, which could have led to type 1 error [54]. The app development process was extensive and included cocreation with the target group and a multidisciplinary research team as well as prepiloting of the demo version in Finnish preschools (see Multimedia Appendix 1 for details). Most intervention arm groups used the app as instructed, suggesting moderate feasibility.

Although the study was able to demonstrate favorable changes in FV acceptance, it also had some limitations that should be addressed. First, our study was not blinded, and the participating early educators in the intervention group knew that the children’s FV acceptance was being measured. The parents of the participating children were also aware of the intervention. However, the app was used in the ECEC centers, whereas parents reported FV acceptance, and thus, the parents did not know exactly how much their children had used the app. In Finland, randomization was conducted at the preschool level to avoid contamination. Owing to nonexistent between-group communication among parents, contamination was not considered probable in Poland. Second, only 72.9% (161/221) of participants had data at both baseline and follow-up. To use data from as many participants as possible, we categorized participants into 3 groups based on their baseline data: missing, lower than median, and median or higher. To determine how the categorization affected the results, we ran multiple sensitivity analyses, which yielded parallel results. Third, as the app was used in a group, we did not know which individual children in the intervention arm groups participated in the game sessions. In addition, as the degree of implementation varied between the ECEC groups, this could have attenuated the observed effects. Fourth, our sample was relatively highly educated, and thus, the results may not be generalizable to socioeconomically disadvantaged groups. Owing to differences in cultural environment, the recruitment process was carried out differently in the 2 countries, which probably resulted in differing participation rates (56% in Finland vs 92% in Poland). Thus, the Polish sample might have been more representative of the target population than the Finnish sample. Furthermore, because of the limited time frame set by the funding period, the intervention was relatively short. As children need repeated taste exposures, preferably integrated with sensory learning strategies as well as nutrition education to get used to different vegetables [55], it is possible that a longer intervention period would have been needed to achieve more prominent and permanent results. However, we realistically aimed to increase FV acceptability, not FV consumption, which would probably require more time. Future studies should include postintervention follow-up to examine the stability of the intervention effects.

### Conclusions

In summary, the Mole’s Veggie Adventures app has the potential to increase FV acceptance among preschoolers. The app can support food education and be incorporated into the preschool curriculum in countries with different educational environments, such as Finland and Poland. When designing serious games for preschoolers, game designers should consider including both home- and preschool-based components to ensure adult endorsement in all relevant environments, which could result in even stronger effects. Future studies should aim to identify the game mechanisms that best support children in making behavior changes.

## Appendix

Multimedia Appendix 1 Detailed description of the application development process.

Multimedia Appendix 2 Video demonstrating the most important sections of the current version of the Mole’s Veggie Adventures application. Note that the version used in the current study differed slightly from the current version.

Multimedia Appendix 3 CONSORT-eHEALTH checklist (V 1.6.1).

## Abbreviations

ECEC: early childhood education and care
EIT: European Institution of Innovation & Technology
FV: fruit and vegetable
