# Supplementary material for: A Mobile App to Increase Fruit and Vegetable Acceptance Among Finnish and Polish Preschoolers: Randomized Trial
Source: JMIR Mhealth Uhealth. 2022 Jan 4;10(1):e30352. doi: 10.2196/30352 (PMC8767468; doi:10.2196/30352)
Supplement: Multimedia Appendix 1 [file mhealth_v10i1e30352_app1.docx]

# Multimedia Appendix 1. Detailed description of the application development process.

# Development of the Mole’s Veggie Adventures application

During 2018 we organized three workshop days in three early childhood education and care (ECEC) centers in Helsinki, Finland. The objectives of the workshops were to learn how children react to tasting vegetables, to test and observe the children’s ability to delay gratification, and to determine which of the existing food-related digital applications were of interest to the children. Based on the experiences collected from the workshops, the game company NordicEdu Oy designed the first version of the prototype in fall 2018. The company then collected feedback from the multidisciplinary research team (experts in nutrition science and early childhood education) and took the proposed modifications into account in order to construct pedagogically and nutritionally evidence-based contents. The prototype was uploaded to the App Store and Google Play for free download.

The first prototype was piloted in 2018 in the same three ECEC centers where the workshops had been organized. The University of Helsinki Ethical Review Board in Humanities and Social and Behavioral Sciences reviewed the pilot study and found it to be ethically acceptable (Statement 28/2018). We recruited children from groups of 5­­–6-year-olds and instructed the groups to use the application as a part of their normal educational routines for 1–3 weeks. After the study period, two researchers (HV & ESk) visited the preschool groups and observed a session in which the group used the application. In two of the ECEC centers, these sessions were video-recorded to facilitate later observation of children’s and early educators’ behavior and reactions from the recordings. In addition, we asked for the opinions of early educators and children about the game.

Based on the information gathered in the prototype piloting, we further developed the prototype into a demo version in collaboration with NordicEdu Oy. The contents of the demo were extended; more vegetables and fruits were added and they were divided into seasons (spring, summer, fall, winter). Based on the information gathered in the DAGIS study (<https://dagis.fi/in-english/>) we chose vegetables that are not frequently used by preschoolers to be introduced in the application (i.e. beans, eggplant, mushrooms). However, in order to not make the application too challenging we also included vegetables and fruits that are commonly well accepted (i.e. tomato, bell pepper, blueberries). Collaborators from the UK and Poland confirmed that the chosen fruits and vegetables were available in their countries as well. The following fruits and vegetables were included in the application:

Winter

- Cabbage
- Peas
- Parsnip
- Green beans and broad beans
- Brussel sprouts
- Pomegranate

Spring

- Bell pepper
- Broccoli
- Spinach
- Asparagus
- Aubergine
- Rhubarb

Summer

- Tomato
- Carrot
- Courgette
- Onion
- Redcurrants
- Plum

Fall

- Beetroot
- Lettuce
- Squash
- Mushrooms
- Kidney, brown and black beans
- Blueberries

In addition, more games aimed at increasing self-regulation skills were added to the demo. All contents were translated from Finnish into English and Polish. We also created a teacher’s guide to support the use of the application in early education. The teacher’s guide is available via the application.

## Using the Mole’s Veggie Adventures

The application is free of charge and can be downloaded to Android- and iOS-based smartphones and tablets from the App Store and Google Play. Originally, the application was designed to be used in groups of 3–10 children and as a part of educational activities in ECEC centers. The application serves as a food education tool and its primary aim is to increase vegetable acceptance among preschoolers. A secondary aim is to support the development and practice of self-regulation skills.

The main elements of the game are the seasons and the six vegetables or fruits linked to each season (see Supplementary Figure 1 for an example). The game environment includes both adult-led sections (e.g. the tasks related to each of the vegetables and fruits included in the application, described in more detail in Supplementary Table 1) and sections that the children can use with or without an adult (Mini-games). The Mini-games are fun activities that can be used to learn new things about vegetables and fruits. The application also features a Taste Bank, which aims at encouraging and inspiring children to taste new vegetables. Guided by an adult, the group can register in the Taste Bank the vegetables and fruits that they have already tasted.


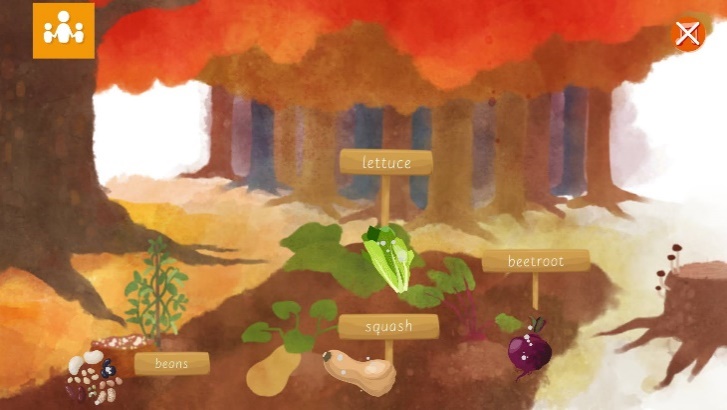

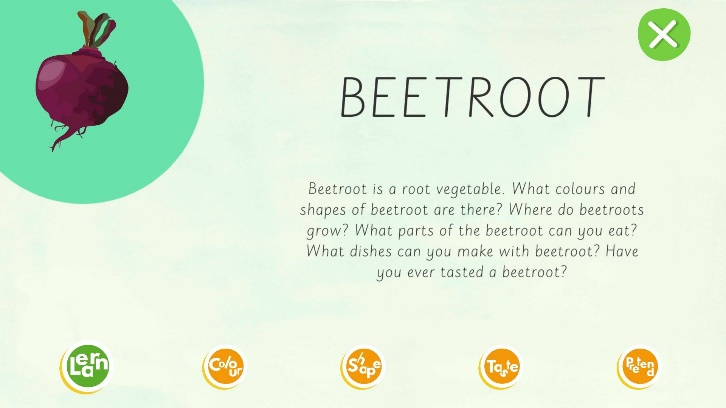


Supplementary Figure 1. An overview of the game environment with four vegetables visible (left) and the Learn task related to the beetroot (right).

Supplementary Table 1. Reasoning behind the tasks and two examples of the tasks.

|  | Idea of the task | Example 1: Courgette | Example 2: Parsnip |
| --- | --- | --- | --- |
| Learn | This task encourages the children to share their knowledge with each other, as the children are instructed to discuss the vegetable or fruit in question. The adult can use the questions shown on the screen to engage a conversation, if needed. Teacher’s guide offers knowledge about the vegetables and fruits included in the application. | Courgettes are vegetables. What colour does it have on the outside? What about inside? What does it look like? How do you eat courgettes? What parts of the courgette can you eat? What dishes can you make with courgettes? When are courgettes picked? Have you ever tasted a raw courgette? What about a cooked one? | Parsnips are root vegetables. Where do parsnips grow? Can you eat raw parsnips? How are parsnips eaten? What dishes can you make with parsnips? Have you ever tasted a parsnip? |
| Color | These tasks guide the children to observe the room in order to find and recognize colors and shapes of fruits and vegetables. | What color are courgettes? Take photos of some things that are the same color as courgettes. | What color are parsnips? Take photos of some things that are the same color as parsnips. |
| Shape |  | What shape are courgettes? Take photos of some things that are the same shape as courgettes. | What shape are parsnips? Take photos of some things that are shaped like a parsnip. |
| Taste | This task challenges the imagination of the children and encourages them to broaden their food-related vocabulary. In addition, actual vegetables and fruits can be used, if applicable. | Pretend that you are eating a courgette. What does the courgette taste like? Take photos of yourselves when you are eating a courgette. Press the record button and say what you think a courgette tastes like. | Pretend that you are eating aparsnip. What does the parsnip taste like? Take photos of yourselves when you are eating a parsnip. Press the record button and say what you think parsnips taste like. |
| Pretend | This task invites the children to physically form the vegetable or fruit in question. The adult can adjust the instructions to fit the particular group of children. | Lie down on the floor on your backs and make yourselves as tall as possible. Then stretch your arms and legs in the same direction (e.g., to the right) and pretend to be long, curved courgettes. Make yourselves tall again and then turn in the other direction, stretching with your arms and legs. You can take a photo. | Pretend you are a parsnip: broaden your shoulders and squeeze legs and feet tightly together. You can line up the parsnips based on their height or the colour of their clothes. You can take a photo. |
| Play | This task contains instructions for a game. The games help learn about new vegetables and fruits as well as practise different skills, such as executive functioning and waiting for one’s turn (self-regulation skills). The adult can adjust all the games to be more appropriate for the age of the group in question and the space and materials available. The teacher’s guide contains detailed instructions and aims for the games. | The adult plays the role of Simon. Simon gives instructions that must be followed if they begin with the phrase “Simon says”. If the instructions do not begin with this phrase, the players should do nothing.  For example, if the adult says “Simon says, hands in the air”, all players should put their hands in the air. If the adult says “Simon says, crouch down”, all players should crouch down. If the adult says “Jump on one leg”, the players must stay still. | The adult plays soothing music, either on tape or, for example, with maracas. The players walk calmly and quietly in the room. When the music stops, they freeze where they are standing like a statue and close their eyes. They must keep their eyes closed until the adult continues to play the music. The game can be made more difficult by lengthening the time the players must remain in the same position. |

## Modifications of the Mole’s Veggie Adventures application

After the completion of the present study, the application has been updated to better fit the home environment. The updates were based on feedback received from the participating ECEC centers and observations made by the research team. The modifications include:

1. The play tasks were moved to form a separate Game Bank and more games suitable for a smaller group were added. The game bank includes a filter function, which can be used to find an appropriate game for a given situation.
2. New Mini-games were created and spoken instructions were added to the Mini-games to allow independent use of the application.
3. The Taste Bank was updated to include three challenges for each week of the year. The first taste challenge includes seasonally harvested vegetables that children have already been introduced to in the Learn section of the application. The second taste challenge includes some other vegetables and fruits with which the children are acquainted. The aim is to taste vegetables from both challenges during the week. The third challenge is normally a little different, e.g. a drawing or a crafts activity. The challenges can be marked as completed and the players can track their progress.
4. The application was translated into Spanish and is now available in four languages: Finnish, English, Polish, and Spanish.
